# Characterization and Evaluation of Antioxidant and Anti-Inflammatory Activities of Flavonoids from the Fruits of *Lycium barbarum*

**DOI:** 10.3390/foods11030306

**Published:** 2022-01-24

**Authors:** Tingting Yang, Yuhang Hu, Yamei Yan, Wangting Zhou, Guijie Chen, Xiaoxiong Zeng, Youlong Cao

**Affiliations:** 1 College of Food Science and Technology, Nanjing Agricultural University, Nanjing 210095, China; 2016208016@njau.edu.cn (T.Y.); 2019108064@njau.edu.cn (Y.H.); 2019208019@njau.edu.cn (W.Z.); guijiechen@njau.edu.cn (G.C.); 2 Institute of Wolfberry Engineering Technology, Ningxia Academy of Agriculture and Forestry Sciences, Yinchuan 750002, China; yanyamei@163.com; 3 National Wolfberry Engineering Research Center, Yinchuan 750002, China

**Keywords:** *Lycium barbarum*, flavonoids, liquid chromatography mass spectrometer (LC-MS), antioxidant, anti-inflammatory

## Abstract

The fruits of *Lycium barbarum* are rich in flavonoids, which may contribute to the health-promoting function of *Lycium barbarum*. However, the composition of flavonoids in the fruits of *Lycium barbarum* (LBFs) has received little attention. Thus, the goal of this work was to identify more kinds of flavonoids from fruits of *Lycium barbarum* by liquid chromatography–mass spectrometry. The potential antioxidant and anti-inflammatory activities of LBFs in vitro were also investigated. Thirteen flavonoid compounds were identified in LBFs, of which daphnetin, 6,7-dihydroxycoumarin, astragalin, taxifolin, eriodictyol, naringenin, and chrysoeriol were identified for the first time in the fruits of *Lycium barbarum*, which greatly enriched the variety of flavonoids in the fruits of *Lycium barbarum*. LBFs showed a similar superior antioxidant activity to vitamin C. Furthermore, LBFs exhibited an anti-inflammatory activity by suppressing the production of nitric oxide and pro-inflammatory cytokines, including tumor necrosis factor-alpha, interleukin-1β, and interleukin-6, in lipopolysaccharide-treated RAW264.7 macrophage cells. This study demonstrated the potential development of LBFs as functional foods.

## 1. Introduction

Flavonoids are a large group of polyhydroxyphenols and widely exist in food-borne plants, such as vegetables, fruits, and grains [1]. Flavonoids occupy the first place in natural phenols and are important secondary metabolites produced in the long-term evolution of plants [2]. These compounds have a variety of pharmacological activities such as antioxidant, antiviral, antitumor, antibacterial, and hypolipidemic activities [3,4,5,6]. In recent years, the concept of promoting human health by the intervening diet has attracted increasingly more attention from researchers [7]. At the same time, natural products have become a research hotspot in topics related to human nutrition and health due to their advantages of small side effects, no drug resistance, and high safety index [8]. However, it is difficult to separate and identify the flavonoids in plant-derived foods, which blocks their further practical applications in functional foods. Thus, the development of a strategy for the identification of flavonoids in different food materials is highly needed.

*Lycium barbarum* is one of the most important traditional Chinese medicines and edible plant species, which is also known as the Goji berry and Chinese wolfberry [9]. The active components of *Lycium barbarum* mainly include polyphenols, pigments, polysaccharides, flavonoids, amino acids, vitamins, and trace elements [10]. Among them, polyphenols, pigments, polysaccharides, and flavonoids are the quantitatively dominant substances in *Lycium barbarum*. In light of the reported research, the main polyphenols from *Lycium barbarum* are gallic acid, catechin, and chlorogenic acid [11]. Carotenoids are a class of natural fat-soluble pigments that exist in *Lycium barbarum*. According to the previous report [12], zeaxanthin dipalmitate is the main constituent of carotenoids in fully ripe fruits of *Lycium barbarum*. The most researched substances in the fruits of *Lycium barbarum* are polysaccharides [13]. In addition, rutin, quercetin, and kaempferol have been reported as the main flavonoids in *Lycium barbarum* [11]. The flavonoids from the fruits of *Lycium barbarum* (LBFs) have gradually become a research hotspot due to their prominent antioxidant, hypolipidemic, hypoglycemic, anti-tumor, and immunity-enhancing activities and their prevention of cardiovascular and cerebrovascular diseases [1]. At present, the research on LBFs mainly focuses on the optimization of extraction, separation, and purification. However, few studies have been performed on the identification and analysis of specific components of LBFs. Recently, a new method for the purification of flavonoids from *Lycium barbarum* with the mixed-mode macroporous adsorption resins (MARs) has been established by computer-assisted calculation of the molecular size of flavonoids and the precise matching of MAR physical and chemical properties [14]. In another study, five flavonoids and three isoflavones were identified in *Lycium barbarum* by HPLC combined with standards [15]. The purpose of the present study, therefore, was to identify more kinds of flavonoids from fruits of *Lycium barbarum*. First, the LBFs were extracted and purified by a resin column in this work. Then, the main flavonoids in LBFs were analyzed by liquid chromatography–mass spectrometry (LC–MS), aiming to provide potential insights into the flavonoids’ composition in *Lycium barbarum*. Furthermore, the potential antioxidant and anti-inflammatory activities in vitro of LBFs were evaluated.

## 2. Materials and Methods

### 2.1. Materials and Reagents

The fresh fruits of *Lycium barbarum* (variety, Ningqi No. 1) were picked in July 2019 from the Wolfberry garden of the National Wolfberry Engineering Research Center (Yinchuan, China; 38°20′ north latitude and 106°16′ east longitude, altitude 2640 m) and dried by gradient hot air at 45–55 °C, affording the dried fruits of *Lycium barbarum*.

The Cell Bank of the Chinese Academy of Sciences (Shanghai, China) provided the murine macrophage RAW264.7 cell line. High-glucose Dulbecco’s modified Eagle’s medium (DMEM), fetal bovine serum (FBS), and penicillin-streptomycin stock solution were purchased from Gibco (Carlsbad, CA, USA). Lipopolysaccharide (LPS) from *Escherichia coli* O111:B4 and 3-(4,5-dimethylthiazol-2-yl)-2,5-diphenyltetrazolium bromide (MTT) were obtained from Sigma-Aldrich Chemical Co., Ltd. (St. Louis, MO, USA). 1,1-Diphenyl-2-picrylhydrazyl (DPPH), reduced nicotinamide adenine dinucleotide (NADH), nitro-tetrazolium blue (NBT) chloride, and phenazine methosulphate (PMS) were purchased from Roche Ltd. (Basel, Switzerland). 2,2’-Azinobis-di-(3-ethyl-benzothiazolin-6-sulfonic acid) diammonium salt (ABTS) and 2,4,6-tris(2-pyridyl)-striazine (TPTZ) were purchased from Aladdin Industrial Inc. (Shanghai, China). ELISA kits for the determination of tumor necrosis factor-alpha (TNF-α), interleukin-6 (IL)-6, and interleukin-1β (IL-1β) were purchased from Neobioscience Biological Technology Co., Ltd. (Shenzhen, China). The kit for the determination of nitric oxide (NO) free radicals was purchased from Beyotime Biotechnology Co., Ltd. (Shanghai, China). All other analytical reagents used in this study were obtained from Sinopharm Chemical Reagent Co., Ltd. (Shanghai, China).

### 2.2. Extraction and Purification of LBFs

The extraction of LBFs was carried out according to the previous report with slight modifications [16]. First, the dried *Lycium barbarum* fruits were ground into 80 mesh powder by a household food mincer (type, JYL-C020E) and extracted with 80% aqueous ethanol solution at 60 °C in triplicate. The extract was concentrated, mixed with 95% aqueous ethanol solution to precipitate the carbohydrates, and centrifuged. The supernatant was then loaded onto an AB-8 macroporous resin column and eluted with 80% aqueous ethanol solution, and the eluent was concentrated and lyophilized to afford LBFs.

In addition, the contents of main components in LBFs were detected by chemical coloration methods, which were the aluminum nitrate-sodium nitrite-sodium hydroxide colorimetric method [17] for the determination of flavonoids, phenol-sulfuric acid method [18] for the determination of total sugars, Coomassie dye binding method [19] for the determination of protein, and Folin–Ciocalteu assay [20] for the determination of total polyphenols.

### 2.3. LC–MS Analysis of LBFs

The extract of LBFs (5.0 mg) was dissolved in 3.0 mL of water containing 0.1% formic acid (FA) and 2% acetonitrile (ACN) for LC–MS analysis [21]. An ACQUITY UPLC HSS T3 column (2.1 mm × 100 mm, 1.8 μm) and a guard column from Waters (Dublin, Ireland) were used. The column oven and auto-sampler temperature were maintained at 40 °C and 10 °C, respectively. Mobile phase A was water containing 0.1% FA (*v/v*), and mobile phase B was ACN. The following linear gradient was used: 0–1.0 min (2% B), 1.0–6.0 min (2–42% B), 6.0–8.0 min (42–65% B), 8.0–10.0 min (65–76% B), 10.0–11.0 min (76–100% B), and 11.0–14.0 min (100–100% B). The flow rate was 0.40 mL/min, and the injector volume of the sample was 5 μL.

For MS analysis, Agilent 1290 II UPLC was coupled with AB Sciex QTOF 5600 Plus (AB Sciex Company, Concord, ON, Canada) and an electrospray ionization (ESI) source under negative ion mode [22]. The MS parameters used for detection were as follows: ESI source voltage, 4.5–5.5 kV; vaporizer temperature, 550 °C; drying gas (N_2_) pressure, 60 psi; atomizer gas (N_2_) pressure, 60 psi; curtain gas (N_2_) pressure, 35 psi; and depolymerization potential, 80 V. The collision energy was 35 ± 15 eV. The scanning range was *m/z* 100–1000. Data acquisition and processing were carried out by using Analyst TF 1.7.1 software. The MS/MS analysis of flavonoids was carried out by the IDA method in information-dependent collection mode [23].

### 2.4. Assay of Antioxidant Capacity

#### 2.4.1. Assay for Scavenging Activity against DPPH Free Radicals

The DPPH free radical scavenging activity was determined by the reported method with slight modifications [15]. Briefly, 25 μL of 0.4 mM DPPH radical solution in ethanol and 100 μL of water were added to 50 μL LBFs or vitamin C (V_C_) solution at different concentrations (25–800 μg/mL). The reaction system was evenly mixed and reacted in the dark at 30 °C for 30 min. The absorbance (Ab*s*) was measured at 517 nm using a Synergy HT microplate reader (Bio-Tek Instruments Inc., Burleigh, Winooski, VT, USA). All the samples were analyzed in three copies. The DPPH free radical scavenging rate was calculated by the following formula:

(1) $$\mathrm{DPPH}\;\mathrm{free}\;\mathrm{radical}\;\mathrm{scavenging}\;\mathrm{rate}\;\left(\%\right)=\frac{\mathrm{Ab}s_{0}-\left(\mathrm{Ab}s_{1}-\mathrm{Ab}s_{2}\right)}{\mathrm{Ab}s_{0}}\times 100$$

Ab*s*_1_ and Ab*s*_0_ are the Ab*s* of the incubated DPPH free radical solution with and without the test substance, respectively; Ab*s*_2_ is the Ab*s* of the sample interference experiment (absolute ethanol instead of DPPH solution).

#### 2.4.2. Assay of Scavenging Activity on Hydroxyl Radicals

The scavenging activity of LBFs on hydroxyl radicals was determined by the method of the report [24] with slight modifications. Briefly, 50 μL of phenanthroline solution (0.75 mM) and 75 μL of phosphate buffer (pH 7.4, 0.15 M) were fully mixed, and then 50 μL of FeSO_4_ solution (0.75 mM) was joined and mixed immediately. Subsequently, the LBF samples with different concentrations (25–800 μg/mL) or V_C_ solutions were added to the reaction system and mixed well. Finally, 50 μL of 0.01% H_2_O_2_ solution was added to start the reaction. It should be noted that every reagent added to the system should be shaken up immediately; otherwise, the local color will be too deep to affect the repeatability of the experiment. Afterward, the reaction was carried out in a water bath at 37 °C for 30 min, and the Ab*s* at 536 nm was determined by a spectrophotometer. The deionized water containing 0.1% H_2_O_2_ was used as a negative control, and PBS was used as a blank control. The scavenging rate on hydroxyl radicals was calculated as follows:

(2) $$\mathrm{Scavenging}\;\mathrm{rate}\;\mathrm{on}\;\mathrm{hydroxyl}\;\mathrm{radical}\;\left(\%\right)=\frac{\mathrm{Ab}s_{2}-A1}{\mathrm{Ab}s_{0}-\mathrm{Ab}s_{1}}\times \;100$$

Ab*s*_0_, Ab*s*_1_, and Ab*s*_2_ are the Ab*s* values of the blank control, negative control, and sample group, respectively.

#### 2.4.3. Assay of Scavenging Activity on Superoxide Anion Radicals

The determination of scavenging activity on superoxide anion radicals was based on the method reported in the literature [25] with slight modifications. NADH, NBT, and PMS were diluted with PBS (0.1 mM, pH 7.4), and 50 μL of LBFs or V_C_ solution (25–800 μg/mL), 50 μL of NADH (468 μM), 50 μL of NBT (156 μM), and 50 μL of PMS (60 μM) were mixed in the 96-well plate and maintained at 25 °C for 10 min, and then the Ab*s* at 560 nm was determined. The scavenging rate on superoxide anion radicals was calculated according to the following formula:

(3) $$\mathrm{Scavenging}\;\mathrm{rate}\;\mathrm{on}\;\mathrm{superoxide}\;\mathrm{anion}\;\mathrm{radicals}\;\left(\%\right)=\frac{\mathrm{Ab}s_{0}-\left(\mathrm{Ab}s_{1}-\;\mathrm{Ab}s_{2}\right)}{\mathrm{Ab}s_{0}}\times 100$$

where Ab*s*_0_ is the Ab*s* of the control (deionized water instead of sample solution), Ab*s*_1_ is the Ab*s* of the sample, and Ab*s*_2_ is the Ab*s* of the sample under the same conditions as Ab*s*_1_ with PBS instead of NBT solution.

#### 2.4.4. Assay for Scavenging Activity on ABTS Radicals

The scavenging activity on ABTS radicals was determined by the ABTS radical cation decolorization method [26]. In a nutshell, the ABTS solution (7.0 mM) was mixed with potassium persulfate (4.95 mM) in equal proportion and stored in a dark place at room temperature for 12 h. The ABTS radical solution was diluted with PBS (0.2 mM, pH 7.4) to a suitable Ab*s* (0.70 ± 0.02) at 734 nm and used as the working solution. Afterward, 20 μL of LBFs or V_C_ with different concentrations was mixed with 200 μL of working solution in a dark place. The Ab*s* of the reaction system was measured at 734 nm. The ABTS free radical scavenging activity was calculated as follows:

(4) $$\mathrm{ABTS}\;\mathrm{free}\;\mathrm{radical}\;\mathrm{scavenging}\;\mathrm{activity}\;\left(\%\right)=\frac{1-\left(\mathrm{Ab}s_{1}-\;\mathrm{Ab}s_{2}\right)}{\mathrm{Ab}s_{0}}\times 100$$

Ab*s*_0_ is the Ab*s* of the control with water instead of the sample, Ab*s*_1_ is the Ab*s* of the sample, and Ab*s*_2_ is the Ab*s* of the sample with only PBS instead of ABTS.

#### 2.4.5. Assay of Total Antioxidant Capacity

After mixing the sample or V_C_ solution of different concentrations with PBS (0.2 M, pH 6.6) and potassium ferricyanide solution (1%, m/v) in the same volume of 50 μL, the mixture was incubated at 50 °C for 20 min. After reaction, the mixture was cooled quickly with running water, and trichloroacetic acid and ferric chloride solutions (50 μL each) were added in turn. The Ab*s* of the mixture was measured at 700 nm [27]. The antioxidant capacity (Ac) was calculated as follows:

Ac = Ab*s*_1_ − Ab*s*_2_ (5)

where Ab*s*_1_ is the Ab*s* of the sample, and Ab*s*_2_ is the Ab*s* of the control with water instead of ferric chloride solution.

#### 2.4.6. Assay for Ferric Reducing Antioxidant Potential (FRAP)

The FRAP was determined according to the reported method [28] with some modifications. FRAP reagent was prepared by mixing 0.3 M acetate buffer (pH 3.6), 10 mM TPTZ, and ferric chloride with the appropriate proportion (10:1:1, *v/v/v*). The FRAP reagent (200 μL) was mixed with 20 μL of sample solution and the reaction system was incubated at 25 °C for 10 min. The Ab*s* at 593 nm was then measured.

### 2.5. Assay of Anti-Inflammatory Activity

#### 2.5.1. Cell Culture and Assay of Cell Viability

The cell culture and assay of cell viability were conducted according to the reported method [29]. In order to analyze the cytotoxicity of LBFs, cell viability was determined by an MTT assay. The cells were cultured in 96-well plates at a density of 5 × 10^4^ cells per well for 12 h, treated with different concentrations of LBFs (10, 20, 50, 100, 150, 200, 300, 400, and 500 μg/mL) for 2 h, and then co-cultured with LPS (final concentration of 1 μg/mL) for 24 h in a 5% CO_2_ incubator at 37 °C. Finally, the Ab*s* at 570 nm was detected by an ELISA microplate reader (BioTeK Instruments, Inc., Winooski, VT, USA). The cell survival rate was calculated as follows:

(6) $$\mathrm{Cell}\;\mathrm{survival}\;\mathrm{rate}\;\left(\%\right)=\frac{\mathrm{Ab}s_{570}\;\mathrm{of}\;\mathrm{treated}\;\mathrm{cells}}{\mathrm{Ab}s_{570}\;\mathrm{of}\;\mathrm{untreated}\;\mathrm{cells}}\times 100$$

#### 2.5.2. Assay of NO Free Radicals

The nitrite concentration in the medium was measured by the Griess method as the index of NO production [3]. Briefly, the RAW264.7 cells (5 × 10^4^/well) were incubated in a 96-well plate for 24 h and treated with 1 μg/mL of LPS after pretreatment with different concentrations of LBFs for 2 h. After co-culture in a 5% CO_2_ incubator at 37 °C, 50 μL of cell culture medium was collected and mixed with 50 μL of Griess reagent I and II. The mixture was incubated at room temperature for 10 min with horizontal shaking, and the Ab*s* of solution was measured at 540 nm by a microplate reader.

#### 2.5.3. Determination of Inflammatory Cytokines

According to the manufacturer’s instructions, the levels of proinflammatory cytokines (TNF-α, IL-1β, and IL-6) in the cell culture medium were measured [30]. In short, the RAW264.7 cells (5 × 10^5^/well) were pretreated with 10–400 μg/mL of LBFs for 2 h before treatment with 1 μg/mL of LPS. After 24 h of culture, the incubation medium of each well was centrifuged (2000× *g*) at 4 °C for 10 min, and the supernatant was taken to determine the levels of TNF-α, IL-1β, and IL-6. Their levels in the solution were calculated according to the standard curves of cytokines. All the experiments were carried out in triplicate.

#### 2.5.4. RNA Extraction and qRT-PCR Analysis

According to the previously reported method, the mRNA expression levels of TNF-α, IL-1β, and IL-6 in macrophages were measured by qRT-PCR [29]. The RAW264.7 cells (5 × 10^5^/well) were inoculated into 6-well plates, after overnight culture; the cells were pretreated with different concentrations of LBFs for 2 h; and they were then treated with 1 μg/mL of LPS for 16 h. The total RNA was extracted from the cells by using the MiniBEST Universal RNA Extraction Kit (TaKaRa Co., Ltd., Beijing, China) according to the manufacturer’s instructions. The concentration and purity of RNA samples were determined by a Nano Drop 2000 spectrophotometer (Thermo Fisher Scientific, Waltham, MA, USA), and the absorbance ratio of all the samples at 260/280 nm was in the range of 1.8–2.0. The qRT-PCR was completed with the Quant Studio^TM^ 6 Flex Real-Time PCR System (ABI, Carlsbad, CA, USA) on utilizing the Power Up™ SYBR^®^ Green Master Mix (ABI, Carlsbad, CA, USA).

The procedure of qRT-PCR amplification was as follows: first, DNA polymerase was activated at 50 °C for 2 min and 95 °C for 2 min, and then the target DNA fragment was amplified for 40 cycles (denaturation at 95 °C for 15 s, annealing at 60 °C, and renaturation with extension for 60 s). Glyceraldehyde-3-phosphate dehydrogenase (GAPDH) was used as an internal reference, and all primer sequences are shown in Table 1.

### 2.6. Statistical Analysis

All the experimental data are shown as means ± standard deviation (*S.D.*) on three independent occasions. To estimate the statistical comparison of data, one-way analysis of variance (ANOVA) or Duncan’s multiple-range test was used based on SPSS 22.0 software, considering *p* < 0.05 to be statistically significant. It is worth noting that the EC_50_ (concentration required to obtain a 50% antioxidant effect) values in the antioxidant assays were also calculated by SPSS 22.0 software with the linear regression equation. All the graphics were made by Origin Pro 2021 SR0 software.

## 3. Results and Discussion

**Figure 1:**
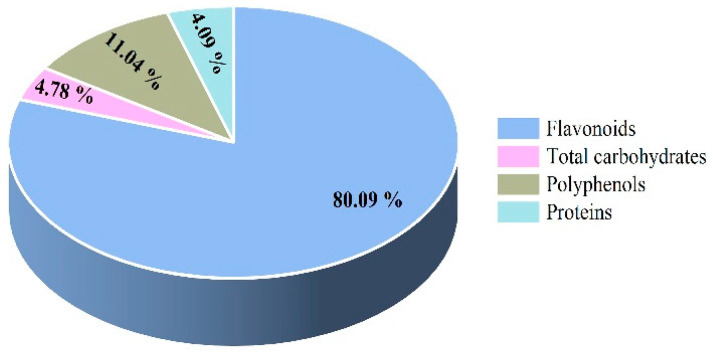
The category and percentage of main components in the extract of flavonoids from the fruits of *Lycium barbarum*.

As shown in Figure 1, the contents of flavonoids, total carbohydrates, polyphenols, and proteins in LBFs were 80.09 ± 0.60%, 4.09 ± 0.63%, 11.04 ± 1.25%, and 4.78 ± 0.82%, respectively.

### 3.1. Identification of Flavonoids by LC–MS

**Figure 2:**
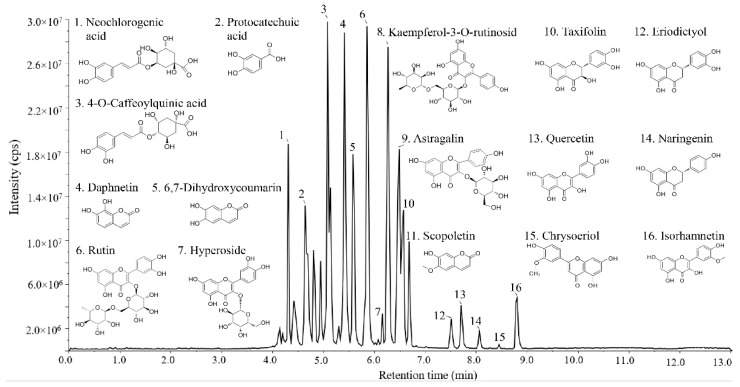
Total ion current chromatogram of sample solution by LC–MS. The names and molecular formulas of 16 compounds identified in the extract of flavonoids from the fruits of *Lycium barbarum*.

Flavonoids are identified as the main phytochemical compounds in *Lycium barbarum*. However, it is difficult to separate and identify the flavonoids in *Lycium barbarum* due to their abundant varieties. In the previous study, ultra-high-performance liquid chromatography with UV detection (UHPLC-UV) was used to analyze the flavonoids in the fruits of *Lycium barbarum*, and five kinds of flavonoids, including (±)-catechin, (−)-epicatechin, rutin, quercitrin, and hesperidin, were identified [1]. Likewise, Zhao et al. [31] identified four flavonoids including kaempferol, quercetin, isorhamnetin, and isoquercetin using matrix-assisted laser desorption/ionization mass spectrometry imaging (MALDI-MSI). However, these methods may omit some kinds of flavonoids. Thus, LC–MS was used to further identify the composition of flavonoids in *Lycium barbarum* in the present study, and the results are shown in Figure 2. Based on the previous reports [32,33,34], sixteen compounds were identified, including neochlorogenic acid (**1**), protocatechuic acid (**2**), 4-*O*-caffeoylquinic acid (**3**), daphnetin (**4**), 6,7-dihydroxycoumarin (**5**), rutin (**6**), hyperoside (**7**), kaempferol-3-*O*-rutinosid (**8**), astragalin (**9**), taxifolin (**10**), scopoletin (**11**), eriodictyol (**12**), quercetin (**13**), naringenin (**14**), chrysoeriol (**15**), and isorhamnetin (**16**) (Table 2). Of these, **6**–**8**, **11**, **13,** and **16** are typical representatives of flavonoids in *Lycium barbarum*, which have been reported before [34,35]. In addition, **4**, **5**, **9**, **10**, **12**, **14,** and **15** were identified for the first time as the flavonoids in *Lycium barbarum*. **9**, a characteristic natural flavone from *Astragalus membranaceus*, has strong antioxidant and anti-inflammatory activities [36]. **10**, also known as dihydroquercetin [37], is a flavonoid extracted from the roots of *Alpine Larix*. It can effectively remove free radicals and toxins in the human body, which is regarded as a precious raw material for the production of food, medicine, and healthcare products. The other compounds such as **1**–**3** are classified into phenolic acids [14,32]. Among these substances, **1** is a natural polyphenolic compound usually found in dried fruits and other plants, and it can inhibit the production of TNF-α and IL-1β, the expression of inducible NO synthase and cyclooxygenase-2, and the activation of phosphorylated NF-κB p65 and p38 MAPK [30]. **2** is a natural polyphenol with neuroprotective effects. Thus, the identified flavonoids in this work greatly enriched the variety of flavonoids in the fruits of *Lycium barbarum*.

### 3.2. Antioxidant Activities of LBFs

**Figure 3:**
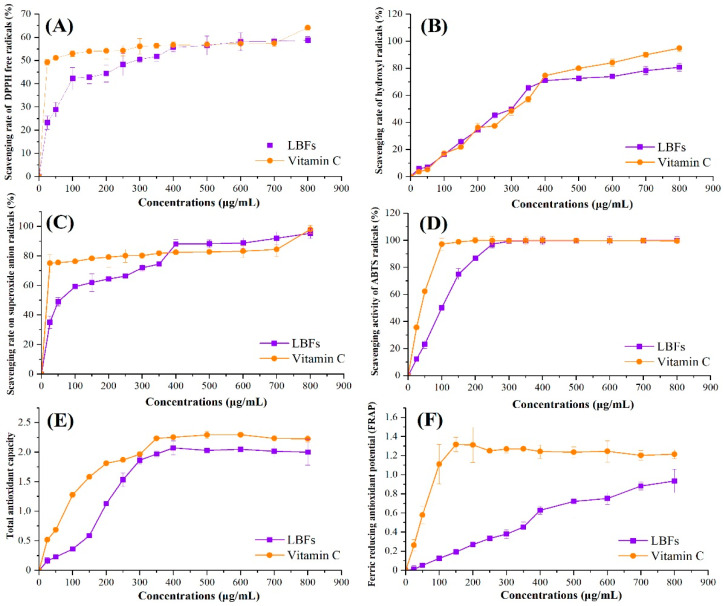
Comparison of antioxidant activities of the extract of flavonoids from the fruits of *Lycium barbarum* and V_C_ determined by DPPH free radical-scavenging assay (**A**), hydroxyl radical-scavenging assay (**B**), superoxide radical-scavenging assay (**C**), ABTS radical-scavenging assay (**D**), total reducing capacity (TRC) assay (**E**), and FRAP assay (**F**). The concentrations of sample solution were 25, 50, 100, 150, 200, 250, 300, 350, 400, 500, 600, 700, and 800 μg/mL in the proper order. Each value was expressed as means ± S.D. (*n* = 3).

The antioxidant activities of LBFs were determined and the results are shown in Figure 3. Compared with V_C_ (positive control), highly concentration-dependent manners of antioxidant activities were found for LBFs at low concentrations (<400 μg/mL). As shown in Figure 3A, when the concentration was over 400 μg/mL, the scavenging rate of LBFs against DPPH radicals was similar to that of V_C_ (55.7 ± 1.9%). In addition, the EC_50_ value was calculated to further quantify the scavenging rate of the sample (Table 3). It is generally believed that the lower the EC_50_ value is, the higher the scavenging ability is. Although the EC_50_ value of V_C_ was much lower than that of LBFs, with the increase in concentration, the clearance rate of LBFs to DPPH free radicals increased significantly to the same level as V_C_, indicating that LBFs showed a strong scavenging activity on DPPH free radicals.

The hydroxyl radical is one kind of important reactive oxygen species (ROS), which has strong oxidizability and is the only oxidant next to fluorine in nature [6]. As shown in Figure 3B, LBFs showed a similar scavenging activity for hydroxyl radicals with that of the positive control at the concentration range of 25–400 μg/mL. When the LBF concentration was higher than 400 μg/mL, the scavenging rate on hydroxyl radicals of LBFs showed a slow downward trend, which was slightly weaker than that of V_C_. At the same time, there was no significant difference in the EC_50_ values between LBFs and V_C_ (Table 3), which further confirmed the results. As shown in Figure 3C, LBFs exhibited a lower scavenging rate of superoxide radicals in the concentrations of 25–350 μg/mL compared with V_C_, whereas the scavenging capacity of LBFs was higher than that of V_C_ (*p* < 0.05), when the concentration of LBFs ranged from 400 to 700 μg/mL. The same trend was also observed in the assay for scavenging activity on ABTS radicals (Figure 3D). When the sample concentration was higher than 300 μg/mL, the scavenging rate of LBFs was almost equal to that of V_C_, and there was no significant difference between the two groups (*p* > 0.05).

Antioxidant activity is directly and positively correlated with reducing capacity [38]. Reducing capacity is usually related to the existence of reducing ketones, which exert an antioxidant effect by providing hydrogen atoms to break free radical chains. The electron-donating ability of a reducing agent is usually determined by the potassium ferricyanide reduction method [11]. Figure 3E shows the total reducing capacities of LBFs and V_C_. The total reducing capacities of all samples increased tightly along with the increase in concentration. When the concentration was 400 μg/mL, the total reducing capacities of LBFs and V_C_ were 2.07 ± 0.12 and 2.25 ± 0.03 (*p* < 0.05), respectively, and remained unchanged with the further increase in concentration.

FRAP is another method to measure the antioxidant activity of samples based on the blue-purple complex formed by ferrous ions and TPTZ under low-pH conditions [28]. As exihibited in Figure 3F, compared with the positive control, the FRAP value of LBFs was in a highly concentration-dependent manner. The FRAP values of LBFs and Vc were 0.63 ± 0.04 and 1.24 ± 0.07 (*p* < 0.05), respectively, when the concentration was 400 μg/mL. Thereafter, as the concentration increased, the FRAP value of Vc gradually decreased, and the rising trend of the FRAP value of LBFs gradually slowed down. When the concentration reached 800 μg/mL, the FRAP values of LBFs and Vc were 0.94 ± 0.12 and 1.22 ± 0.05, respectively.

In conclusion, LBFs exhibited antioxidant activities, including superior scavenging activities on DPPH, hydroxyl, superoxide ABTS radicals, and a noticeable ferric reducing antioxidant. It has been reported that the components of 80% ethanol extracts were considered to have the greatest contribution to the antioxidant activity of *Lycium* *barbarum* [10]. Based on the previous work and results in this work, the main components of *Lycium* *barbarum* extracted with 80% aqueous ethanol solution were flavonoids, except a small amount of other polyphenols. It has also been pointed out in other research on the chromatographic determination and antioxidant activity evaluation of phenolic acids and flavonoids in *Lycium* *barbarum* that the flavonoid components such as rutin, quercetin, and naringenin showed a high effect on scavenging DPPH free radicals [1]. The studies of flavonoids in strawberry, blueberry, and chokeberry also showed similar antioxidant activities as *Lycium barbarum* flavonoids with reducing ability and radical scavenging activity [39,40]. In addition, the analysis of the antioxidant components and total antioxidant capacity of goji berries confirmed that the scavenging ability and the ability to prevent the formation of free radicals were closely related to the concentration and composition of flavonoids of *Lycium* *barbarum* [41]. Thus, it was speculated that flavonoids might be the important active components contributing to the antioxidant activity of *Lycium* *barbarum*.

### 3.3. Anti-Inflammatory Activity of LBFs

Epidemiological studies have shown that excessive production of proinflammatory factors may lead to the development of chronic inflammatory diseases, such as cancer, inflammatory bowel disease, and metabolic syndrome [42]. The mouse macrophage RAW264.7 cell line is generally considered as a suitable cell model for the in vitro study of anti-inflammatory diseases [43]. LPS is the main component of the cell wall of Gram-negative bacteria, which is widely used to establish the macrophage inflammation model in vitro [44]. In this study, the inflammatory model of RAW264.7 macrophages stimulated by LPS was used to investigate the anti-inflammatory activity of LBFs.

#### 3.3.1. Effects of LBFs on Cell Viability

**Figure 4:**
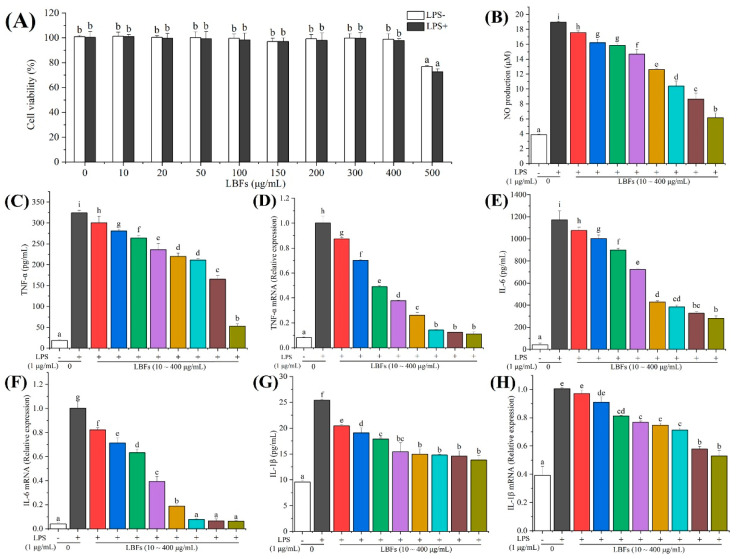
Cytotoxic effects on RAW264.7 cells and effects on LPS-induced pro-inflammatory cytokine production and mRNA expression of the extract of flavonoids from the fruits of *Lycium barbarum*. In cytotoxicity test (**A**), cells were pretreated with different concentrations (10, 20, 50, 100, 150, 200, 300, and 400 μg/mL) of LBFs for 2 h and then treated with 1 μg/mL of LPS for 24 h. (**B**) The NO level in the cell culture was detected by Griess assay. In the anti-inflammatory activity test, RAW264.7 cells were pretreated with different concentrations of LBFs for 2 h and then coexisted with LPS for 24 h. The culture supernatant was collected, and the levels of TNF-α (**C**), IL-6 (**E**), and IL-1β (**G**) were detected by ELISA kits. At the same time, cellular lysate was added into the culture well to extract total mRNA, and then the mRNA expression levels of TNF-α (**D**), IL-6 (**F**), and IL-1β (**H**) were determined by qRT-PCR. Each value is shown as means ± S.D. (*n* = 3). Data with diverse letters are significantly different (*p* < 0.05).

The safety evaluation should be carried out before the development of LBFs for functional foods. Thus, an MTT assay was used to evaluate the effect of LBFs on the viability of RAW264.7 macrophages [29]. In view of the concentration-dependent manner of LBFs in the antioxidant experiment, the cells were pretreated with 10–500 μg/mL of LBFs for 2 h and then treated with 1 μg/mL of LPS for 24 h. As shown in Figure 4A, LBFs at the concentration of 10–400 μg/mL showed limited effect on cell viability with and without the addition of LPS. When the concentration of LBFs was at 500 μg/mL, the cell viability was significantly reduced to 72.71 ± 2.39% (*p* < 0.05) compared with the blank control. Therefore, LBFs at the concentration of 10–400 μg/mL had no cytotoxicity on RAW264.7 cells, which was used for the subsequent experiments.

#### 3.3.2. Effects of LBFs on the Production of NO

NO, a short-lived free radical produced in a wide variety of cells, has various biological functions in response to inflammatory stimuli, such as LPS, which is regarded as an indicator of inflammatory response. To evaluate the effect of LBFs on the production of LPS-induced NO in RAW264.7 cells, the level of NO in the culture medium was detected by Griess assays. As shown in Figure 4B, the level of NO in macrophages was significantly increased by treatment with 1 μg/mL of LPS (*p* < 0.05) compared with the blank control. Nevertheless, pretreatment with LBFs could noticeably decrease the LPS-induced NO production in a dose-dependent manner. Especially, the production of NO decreased to 6.15 ± 0.58 μM after pretreatment with LBFs at 400 μg/mL. These results indicated that LBFs exihibited anti-inflammatory activity by the inhibition of NO production in a remarkably concentration-dependent manner.

#### 3.3.3. Effects of LBFs on the Production of LPS-Induced Pro-Inflammatory Cytokines and mRNA Expression

Macrophages are widely distributed in the human body and play a key role in the immune system by providing an immediate defense against foreign pathogens. LPS is one of the most powerful activators of macrophages, as we know [4]. The activation of macrophages by LPS is the result of toll-like receptor-4 (TLR-4)-mediated intracellular signaling cascades, and ultimately leads to the expression of proinflammatory mediators, such as TNF-α, IL-6, and IL-1β [45]. TNF-α is the earliest and most important inflammatory mediator in the process of inflammatory reaction, which can activate neutrophils and lymphocytes, increase the permeability of vascular endothelial cells, regulate the metabolic activity of other tissues, and promote the synthesis and release of other cytokines [43]. Both IL-6 and IL-1β belong to IL, which is a kind of cytokine of the chemokine family, and they can induce B-cells to differentiate and produce antibodies and T-cells to proliferate and differentiate. They participate in the immune response of the body and are the promoters of inflammatory reaction [44].

In the present study, the effects of LBFs on the LPS-induced production of TNF-α, IL-6, and IL-1β were investigated by ELISA. The results indicated that the levels of TNF-α, IL-6, and IL-1β remarkably increased from 18.54 ± 1.86 to 324.11 ± 6.13 pg/mL (Figure 4C), 42.22 ± 14.09 to 1172.36 ± 80.73 pg/mL (Figure 4E), and 9.53 ± 0.35 to 25.41 ± 0.23 pg/mL (Figure 4G), respectively, after a 24 h intervention with LPS alone compared with the blank control. In addition, the inhibitory effects of LBFs pretreatment on the LPS-induced production of TNF-α, IL-6, and IL-1β were observed in a dose-dependent manner. Especially, when the pretreatment of LBFs was at 400 μg/mL, the LPS-induced levels of TNF-α, IL-6, and IL-1β declined to 52.62 ± 5.46, 280.13 ± 21.79, and 13.84 ± 0.88 pg/mL, respectively.

Furthermore, the mRNA expression levels of TNF-α, IL-6, and IL-1β in RAW264.7 cells were investigated, and the results showed that the mRNA expression levels of TNF-α, IL-6, and IL-1β significantly increased after LPS-stimulation in the RAW264.7 macrophage cells (*p* < 0.05), which is consistent with the previous work [29,44]. As expected, the mRNA expression levels of TNF-α, IL-6, and IL-1β decreased by preconditioning with different concentrations (10–400 μg/mL) of LBFs (Figure 4D,F,H). Especially, the mRNA expression levels of TNF-α, IL-6, and IL-1β decreased to 11.01 ± 1.69%, 6.37 ± 0.3%, and 53.04 ± 3.77% after treatment of LBFs at 400 μg/mL, respectively, compared with those of the cells acitivated with LPS alone.

In the process of inflammation, high levels of IL-1β and IL-6 can lead to fever, hypotension, immune disorder, and chronic inflammation, while overexpression of NO and TNF-α can promote T-cells to produce various inflammatory cytokines, including IL-1β and IL-6, and then stimulate the occurrence of an inflammatory response [3]. In this study, RAW264.7 cells were stimulated by LPS to produce excessive inflammatory mediators of NO and TNF-α, and proinflammatory cytokines of IL-1β and IL-6. However, the levels of inflammatory mediators (NO and TNF-α) and cytokines (IL-1β and IL-6) in RAW264.7 cells decreased significantly in a dose-dependent manner after intervention with LBFs. In conclusion, LBFs showed a good anti-inflammatory effect by inhibiting the excessive secretion of inflammatory mediators and inflammatory factors in the process of inflammation. These results suggested that the decrease in TNF-α, IL-6, and IL-1β production in a concentration-dependent manner by LBFs might be related to the transcriptional inhibition of the genes of TNF-α, IL-6, and IL-1β.

Existing studies have shown that baicalein exerts its anti-inflammatory effect by inhibiting mitogen-induced T-cell activation, proliferation, and cytokine secretion [46]. Anti-oxidative assays as markers for the anti-inflammatory activity of flavonoids showed that with the increase in flavonoids (quercetin, hyperoside, eriodictyol, naringenin, isorhamnetin, etc.) at different concentrations (10, 20, and 50 mg/L), the gene expression levels of inflammatory factors such as IL-1β, IL-6, IL-8, and TNF-α were remarkably decreased [47]. Moreover, the study of antioxidant and anti-inflammatory activities of 100 pure compounds pointed out that kaempferol and quercetin could effectively inhibit the secretion of IL-6, IL-1β, and TNF-α. Quercetin (10–25 mol/L) could reduce the contents of NO and TNF-α in mouse glioma cells induced by LPS [48]. In the study of the regulating pathway by astragalin to endotoxin-induced oxidative stress [36], astragalin inhibits endotoxin-induced oxidative stress by interfering with the TLR-4-PKCβ2-NADPH oxidase signal pathway, improving epithelial eosinophilia and apoptosis related to oxidative stress, and reducing the levels of inflammatory factors and their gene expression of inflammation. Thus, LBFs showed superior antioxidant and anti-inflammatory activities, which might be responsible for the health-promoting functions of *Lycium barbarum*. However, the antioxidant and anti-inflammatory activities of flavonoids in vivo are still unknown, which should be further investigated by animal models. Furthermore, the specific component responsible for the bioactivities of flavonoids from *Lycium barbarum* is still unknown; thus, we should separate and purify each flavonoid in LBFs and evaluate the bioactivity in the future. This work demonstrated the potential bioactivity of flavonoids from *Lycium barbarum* in vitro, which may promote the further works on bioactivities in vivo of flavonoids from the fruits of *Lycium barbarum*.

## 4. Conclusions

In the present study, thirteen flavonoids in LBFs were identified by LC–MS. Among them, seven kinds of flavonoids (daphnetin, 6,7-dihydroxycoumarin, astragalin, taxifolin, eriodictyol, naringenin, and chrysoeriol) were identified in *Lycium barbarum* for the first time. In the aspect of antioxidant activity, LBFs showed a strong free radical scavenging ability and reducing capacity in a dose-dependent manner. Moreover, LBFs pretreatment in the LPS-induced macrophages model could reduce the inflammatory response by inhibiting the production of inflammatory mediators and cytokines. The results suggested the potential antioxidant and anti-inflammatory activities of LBFs, providing the theoretical foundation and experimental evidence for the further applications of *Lycium* *barbarum* in food and pharmaceutical industries.

## Figures and Tables

**Table 1:** Sequences of PCR primers used for qRT-PCR.

Target Gene	Primer Sequence	Annealing T (°C)
TNF-α	FW: CTCATGCACCACCATCAAGG	60
	RV: ACCTGACCACTCTCCCTTTG	
IL-1β	FW: AGCTTCAAATCTCGCAGCAG	59
	RV: TCTCCACAGCCACAATGAGT	
IL-6	FW: CTCTGGCGGAGCTATTGAGA	60
	RV: AAGTCTCCTGCGTGGAGAAA	
GAPDH	FW: GGACTTACAGAGGTCCGCTT	59
	RV: CTATAGGGCCTGGGTCAGTG	

**Table 2:** Identification of flavonoids in the fruits of *Lycium barbarum*.

No.	Molecular Formula	Theoretical *m/z* [M − H]	Retention Time	Component Name
1	C_16_H_18_O_9_	353.309	4.31	Neochlorogenic acid
2	C_7_H_6_O_4_	153.120	4.64	Protocatechuic acid
3	C_16_H_18_O_9_	353.309	5.08	4-*O*-Caffeoylquinic acid
4	C_9_H_6_O_4_	177.140	5.40	Daphnetin
5	C_9_H_6_O_4_	177.141	5.58	6,7-Dihydroxycoumarin
6	C_27_H_30_O_16_	609.518	5.86	Rutin
7	C_21_H_20_O_12_	463.376	6.16	Hyperoside
8	C_27_H_30_O_15_	593.518	6.26	Kaempferol-3-o-rutinosid
9	C_21_H_20_O_11_	447.377	6.51	Astragalin
10	C_15_H_12_O_7_	303.252	6.56	Taxifolin
11	C_10_H_8_O_4_	191.168	6.72	Scopoletin
12	C_15_H_12_O_6_	287.252	7.65	Eriodictyol
13	C_15_H_10_O_7_	301.236	7.72	Quercetin
14	C_15_H_12_O_5_	271.253	8.07	Naringenin
15	C_16_H_12_O_6_	299.263	8.36	Chrysoeriol
16	C_16_H_12_O_7_	315.262	8.79	Isorhamnetin

**Table 3:** EC_50_ values of antioxidant activities of the extract of flavonoids from the fruits of *Lycium barbarum* (LBFs) and V_C_ determined by DPPH free radical scavenging assay, hydroxyl radical (HR) scavenging assay, superoxide radical (SR) scavenging assay, and ABTS radical scavenging assay. Data are shown as the means ± S.D. Data bearing different letters are significantly different (*p* < 0.05).

	LBFs	V_C_
EC_50/DPPH_ (mg/mL)	294.864 ± 2.470 ^b^	40.436 ± 1.607 ^a^
EC_50/HR_ (mg/mL)	275.048 ± 2.439 ^a^	265.520 ± 2.424 ^a^
EC_50/SR_ (mg/mL)	66.463 ± 1.820 ^b^	1.919 ± 0.283 ^a^
EC_50/ABTS_ (mg/mL)	82.663 ± 1.917 ^b^	34.348 ± 1.536 ^a^

## Data Availability

The data presented in this study are available in the article.
